# Simulation of Percolation Threshold, Tunneling Distance, and Conductivity for Carbon Nanotube (CNT)-Reinforced Nanocomposites Assuming Effective CNT Concentration

**DOI:** 10.3390/polym12010114

**Published:** 2020-01-05

**Authors:** Yasser Zare, Kyong Yop Rhee

**Affiliations:** Department of Mechanical Engineering, College of Engineering, Kyung Hee University, Yongin 446-701, Korea; y.zare@aut.ac.ir

**Keywords:** polymer nanocomposites, conductivity, tunneling effect, percolation threshold, interphase

## Abstract

This article suggests simple and new equations for the percolation threshold of nanoparticles, the tunneling distance between nanoparticles, and the tunneling conductivity of polymer carbon nanotubes (CNTs) nanocomposites (PCNT), assuming an effective filler concentration. The developed equations correlate the conductivity, tunneling distance, and percolation threshold to CNT waviness, interphase thickness, CNT dimensions, and CNT concentration. The developed model for conductivity is applied for some samples and the predictions are evaluated by experimental measurements. In addition, the impacts of various parameters on the mentioned terms are discussed to confirm the developed equations. Comparisons between the calculations and the experimental results demonstrate the validity of the developed model for tunneling conductivity. High levels of CNT concentration, CNT length, and interphase thickness, as well as the straightness and thinness of CNTs increase the nanocomposite conductivity. The developed formulations can substitute for the conventional equations for determining the conductivity and percolation threshold in CNT-reinforced nanocomposites.

## 1. Introduction

Polymer carbon nanotubes (CNTs) nanocomposites (PCNT) are very attractive from research and application points of view, because they can elucidate important properties when the concentration of nanoparticles reaches the percolation threshold, which is the minimum CNT content that produces the conductive networks in nanocomposites [1,2,3,4,5,6,7,8,9,10,11,12,13,14,15]. Long and thin CNTs normally introduce a low percolation threshold, producing a conductive nanocomposite by incorporation of very low CNT content [16]. Accordingly, CNTs are a desirable nanofiller for development of advanced nanocomposites with improved properties.

Many parameters affect the conductivity of polymer nanocomposites. The main effects are attributed to the characteristics of conductive nanoparticles such as content, dimensions, conductivity, and dispersion quality [17,18,19]. Additionally, the size and density of conductive networks largely manipulate the conductivity of nanocomposites, because the networks provide the conductive paths for charge transfer. Moreover, the immense surface area of nanoparticles per unit volume creates an intermediate phase between the polymer matrix and the nanoparticles, called the interphase, which governs the behavior of nanocomposites [20,21]. The roles of the interphase in the mechanical performance of polymer nanocomposites have been widely analyzed by experimental and theoretical methods [22,23,24]. Moreover, the interphase zones can form the connected structures in polymer nanocomposites, which lower the percolation threshold [25,26]. Thus, the interphase areas definitely influence the electrical conductivity of nanocomposites by reducing the percolation threshold, although this matter has been relatively little discussed in the literature.

Electron tunneling as the main mechanism for conductivity of PCNT includes the transferring of electrons between nearby nanotubes (tunneling spaces) based on quantum mechanics [27,28]. As a result, the tunneling effect does not involve the attached nanotubes, and the nearby CNTs can transfer the charges in nanocomposites. However, electrons can be transported via tunneling zones when the separation distance between nanotubes is small enough. Accordingly, the tunneling effect mostly depends on the distance between neighboring CNTs [29,30]. Only a few studies have focused on the tunneling mechanism in PCNT [31,32]. For example, Feng and Jiang [31] considered the interphase layer surrounding CNTs as a tunneling area and developed various equations for tunneling distance, interphase thickness, and conductivity. Generally, the extant studies on the tunneling conductivity show unclear and multifaceted terms and equations that are rarely applied in practice. Moreover, the available models inadequately reflect the influences of filler and interphase dimensions on the tunneling properties and conductivity. In particular, although filler size generally expresses the percolation threshold of nanoparticles, the roles of tunneling distance and the interphase layer have been ignored in the previous articles [33,34].

We have published some reports on the conductivity of PCNTs assuming the interphase zone surrounding CNTs and the tunneling region between adjacent CNTs [35,36,37,38]. Those studies considered a constant value for the tunneling distance (at different filler concentrations), and expressed the percolation threshold as a function of CNT dimensions and interphase thickness. In this paper, we join two exponential equations for the electrical conductivity of nanocomposites to derive the proper equations for the tunneling distance and percolation threshold. We consider the effective CNT concentration, which includes the concentrations of both CNTs and interphase zone. Moreover, we express the tunneling distance and percolation threshold by CNT concentration, CNT dimensions, as well as the interphase thickness around the CNTs. Additionally, we develop the exponential equation suggested by Ambrosetti et al. [39] for the electrical conductivity of nanocomposites using the mentioned terms. The developed equation suggests the electrical conductivity by CNT waviness, interphase thickness, CNT size, and effective CNT volume fraction. The estimates of electrical conductivity by the developed equation are compared to the various experimental results from valid literature. In addition, the impacts of various parameters on the mentioned terms are analyzed to confirm the correctness of the established equations.

## 2. Methodology

A simple model was suggested for the tunneling electrical conductivity of polymer nanocomposites [40] as:

(1) $$\sigma =\sigma_{0}\exp(-\frac{2d}{z})$$

where *σ*_0_ is a parameter, *d* is the tunneling distance, and *z* is the characteristic tunneling length. This model has been widely applied in different studies on the conductivity of polymer nanocomposites, especially PCNT [39,40,41].

Ambrosetti et al. [39] developed this model for different nanocomposites, and suggested the conductivity in nanocomposites containing cylindrical particles as:

(2) $$\sigma =\sigma_{0}\exp(-\frac{4.8R^{2}}{z\varphi_{f}^{}l})$$

where *R*, $\varphi_{f}$, and *l* denote the radius, volume fraction, and length of the nanoparticles, respectively.

Comparing Equations (1) and (2) can suggest the following equation for the *d* parameter as:

(3) $$d=\frac{2.4R^{2}}{\varphi_{f}^{}l}$$

The tunneling distance between adjacent CNTs in the conductive networks can be expressed [40] by:

(4) $$d=A\varphi_{f}^{-1/3}$$

where *A* is a constant parameter. Similarly, there is a maximum separation distance between CNTs allowing the tunneling effect (*d_m_*), which suggests [31]:

(5) $$A=d_{m}\varphi_{p}^{1/3}$$

By replacing *A* from Equation (5) into Equation (4), the tunneling distance can be given by:

(6) $$d=d_{m}\frac{\varphi_{p}^{1/3}}{\varphi_{f}^{1/3}}$$

By joining Equations (3) and (6), it is possible to express the percolation threshold assuming a tunneling distance as:

(7) $$\varphi_{p}^{}={\left(\frac{2.4R^{2}}{ld_{m}\varphi_{f}^{2/3}}\right)}^{3}$$

**Figure 1:**
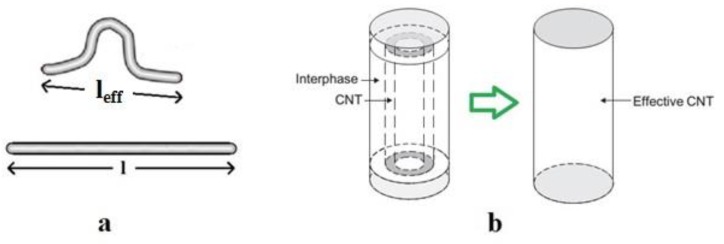
(**a**) Wavy and straight carbon nanotubes (CNTs), and (**b**) effective nanotube assuming an interphase (reproduced from [31], with permission from Elsevier, 2019).

However, the exceptional length of CNTs commonly causes waviness in polymer nanocomposites [42]. The effective length of nanotubes (*l_eff_*) (Figure 1a) can be assumed by the waviness parameter as:

(8) $$u=\frac{l}{l_{eff}}$$

where *u* = 1 and *u* > 1 denote no waviness (straight CNTs) and more waviness, respectively. Thus, the effective length of nanotubes is presented as:

(9) $$l_{eff}=\frac{l}{u}$$

Moreover, the interphase regions have a positive effect on the conductivity of nanocomposites by reduction of the percolation threshold and the growth of networked structures [43]. In fact, the interphase regions and waviness modify the effective volume fraction of CNTs in nanocomposites (Figure 1b). The effective volume fraction in CNT nanocomposites [31] can be given by:

(10) $$\varphi_{eff}=\frac{{\left(R+t\right)}^{2}(l/u+2t)}{R^{2}l/u}\varphi_{f}$$

where *t* is the interphase thickness.

Assuming the roles of the interphase and waviness by Equations (9) and (10), the conductivity, tunneling distance, and percolation threshold are suggested as:

(11) $$\sigma =\sigma_{0}\exp(-\frac{4.8uR^{2}}{z\varphi_{eff}^{}l})$$

(12) $$d=\frac{2.4uR^{2}}{\varphi_{eff}^{}l}$$

(13) $$\varphi_{p}^{}={\left(\frac{2.4uR^{2}}{ld_{m}\varphi_{eff}^{2/3}}\right)}^{3}$$

which express the influences of waviness, interphase thickness, filler dimensions, and effective filler fraction on the mentioned terms.

## 3. Results and Discussion

### 3.1. Electrical Conductivity

We applied the suggested equations to calculate the conductivity at different levels of the material and interphase parameters. Moreover, we compared the calculations of electrical conductivity to the experimental measurements in some samples to demonstrate the predictability of the developed model.

**Figure 2:**
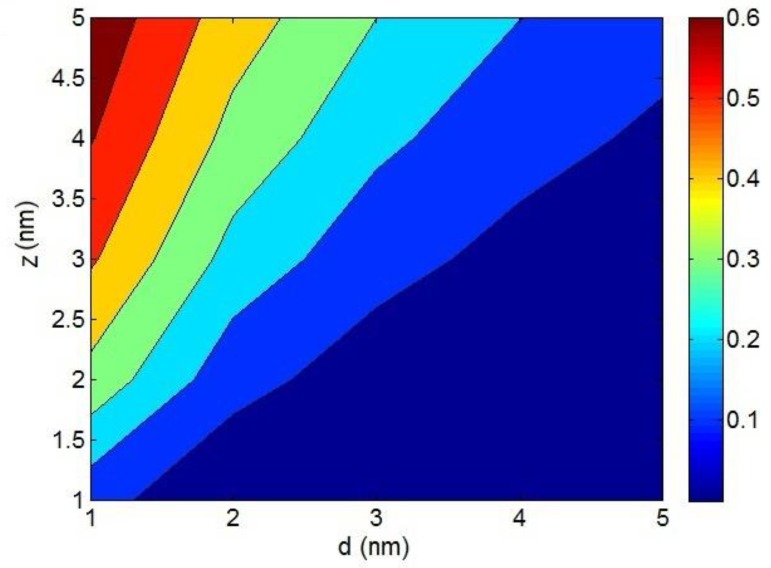
Contour plot for the roles of the *d* and *z* parameters in the conductivity of nanocomposites at *σ*_0_ = 1 S/m by Equation (1).

Figure 2 illustrates the effects of the d and *z* parameters on the conductivity by a contour plot at *σ*_0_ = 1 S/m. The highest conductivity is calculated by the smallest d and the highest *z*. As observed, σ = 0.6 S/m is obtained at *d* = 1 nm and *z* = 5 nm. However, an insulating effect is observed at high d and low *z*. Therefore, a desirable conductivity is achieved by a short tunneling distance and high characteristic tunneling length. These results were expected because a short tunneling distance and a large characteristic tunneling length effectively amplify the tunneling effect, while a long tunneling distance and a small characteristic tunneling length weaken the tunneling mechanism.

**Figure 3:**
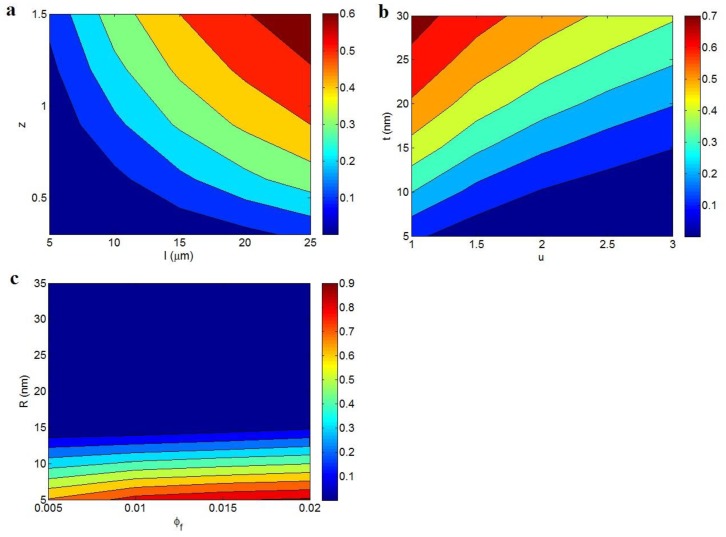
The electrical conductivity of nanocomposites at different ranges of (**a**) the *l* and *z*, (**b**) *u* and *t*, and (**c**) $\varphi_{f}$ and *R* parameters based on Equation (11).

Figure 3 depicts the electrical conductivity as a function of different parameters according to Equation (11). Figure 3a exhibits the effects of the *l* and *z* parameters on the conductivity at average = 0.01, *R* = 10 nm, *t* = 10 nm, and *u* = 1.3. The highest conductivity is observed at the highest values of the *l* and *z* parameters, while the low ranges of these parameters significantly diminish the conductivity. As a result, both the *l* and *z* parameters, being the CNT length and characteristic tunneling length, respectively, directly affect the tunneling conductivity of nanocomposites. In other words, long nanotubes and a high *z* value produce high conductivity in nanocomposites. Long nanotubes have good potential for connecting and networking because they have more contacts compared to short nanotubes. Moreover, the conductive networks produced by long nanotubes can cover a large area in the nanocomposite, which can positively affect the conductivity [44]. Accordingly, long nanotubes are necessary for large networks and high conductivity. It should be noted that the conductivity directly depends on the characteristic tunneling length, but this parameter has not been precisely defined to date.

Figure 3b illustrates the variation of conductivity at dissimilar ranges of the *u* and *t* parameters and average = 0.01, *R* = 10 nm, *l* = 10 μm, and *z* = 1. A high *u* value and small *t* value result in an insulated nanocomposite, while the lowest *u* and the highest *t* produce the best conductivity. These evidences show that the waviness and interphase thickness inversely and directly affect the conductivity, respectively. Therefore, a desired conductivity is obtained by low waviness and a thick interphase, while great waviness and a thin interphase cannot improve the conductivity of nanocomposites. The waviness lowers the effective length and conductivity of CNTs in the nanocomposites [42]. The waviness actually worsens the percolation threshold of nanotubes and the characteristics of conductive networks, leading to poor conductivity. The detrimental effects of waviness on the conductivity and mechanical performance of CNT nanocomposites were reported in previous articles [45,46]. Therefore, the inverse dependency of conductivity on the CNT waviness is sensible and confirms our new equation.

A thick interphase increases the effective volume fraction of the nanoparticles in nanocomposites based on Equation (10), and thus it can decrease the tunneling distance between neighboring CNTs (Equation (12)) and lower the percolation threshold (Equation (13)). As a result, a thicker interphase produces a shorter tunneling distance between CNTs and larger networks in nanocomposites, which considerably raises the conductivity, as predicted by the model developed here. The positive impacts of interphase zones on the percolation threshold of CNTs and the mechanical properties of nanocomposites have been addressed in the literature [22,25], but their influence on the conductivity has not yet been explained.

The effects of $\varphi_{f}$ and *R* on the conductivity are also depicted in Figure 3c at average *l* = 10 μm, *t* = 10 nm, *u* = 1.3, and *z* = 1. A high *R* decreases the conductivity to about 0, whereas the highest conductivity is obtained at the highest $\varphi_{f}$ and the smallest *R*. Therefore, a high concentration of thin nanotubes causes a desirable conductivity, whereas the various concentrations of thick CNTs result in a low conductivity. The conductivity of CNTs was reported as about 106 S/m, which is about 1021 times that of polymer conductivity [18]. Thus, the conductivity of nanocomposites is controlled by the concentration of CNTs because polymer matrices are generally insulated. Moreover, thin CNTs increase the effectiveness of the nanoparticles in nanocomposites because they produce a short tunneling distance and a low percolation threshold, according to Equation 12 and 13. Therefore, the developed model demonstrates the correct dependencies of conductivity on filler concentration and radius. The same outputs were also proposed by previous studies approving the present results [47].

**Figure 4:**
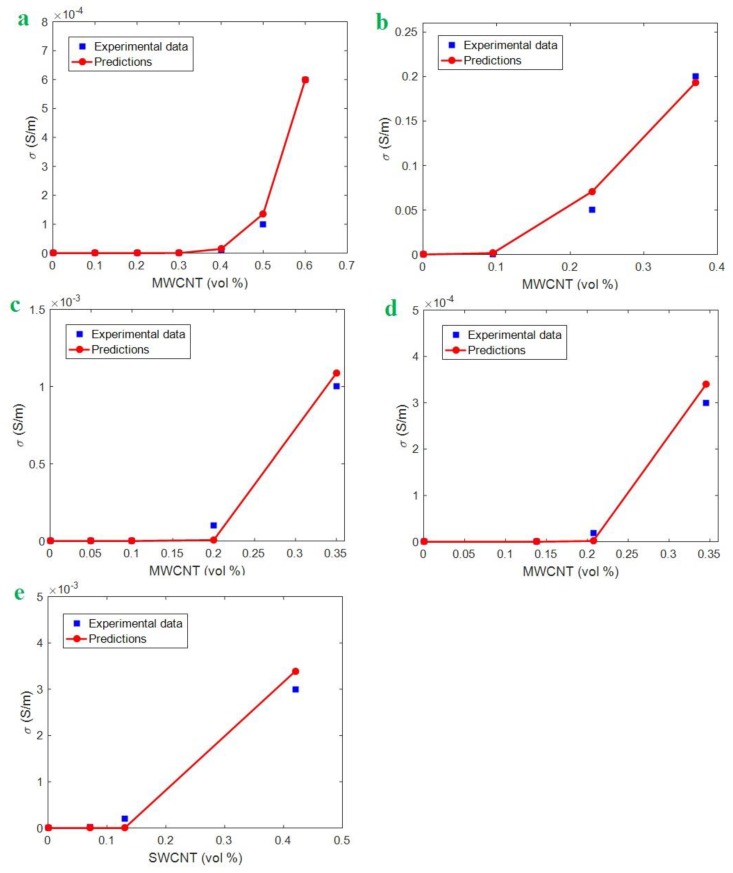
Experimental results of conductivity and the predictions of the developed model (Equation 11) for (**a**) polydimethylsiloxane (PDMS)/multi-walled CNT (MWCNT) [48], (**b**) ultra-high-molecular-weight polyethylene (UPE)/MWCNT [49], (**c**) poly (vinyl chloride) (PVC)/MWCNT [50], (**d**) poly (ethylene terephthalate) (PET)/MWCNT [51], and (**e**) epoxy/single-walled CNT (SWCNT) [52] nanocomposites.

We chose five samples from the literature for analysis of the developed model: Polydimethylsiloxane (PDMS)/multi-walled CNT (MWCNT) (*R* = 5 nm, *l* = 15 μm, *u* = 1.2) [48]; ultra-high-molecular-weight polyethylene (UPE)/MWCNT (*R* = 8 nm, *l* = 8 μm, *u* = 1.2) [49]; poly (vinyl chloride) (PVC)/MWCNT (*R* = 8 nm, *l* = 16 μm, *u* = 1.2) [50]; poly (ethylene terephthalate) (PET)/MWCNT (*R* = 5 nm, *l* = 1 μm, *u* = 1.2) [51]; and epoxy/single-walled CNT (SWCNT) (*R* = 1 nm, *l* = 2 μm, *u* = 1.6) [52]. Figure 4 presents the predictions of the developed model and the experimental results for these reported samples. The good agreements between the experimental results and the predictions in all the samples illustrate that our new model can accurately estimate the conductivity of PCNT. These plots are the best fitting of calculations on the experimental data using the developed model. Some insignificant deviations are observed between the experimental data and model outputs, especially at high CNT concentrations, due to the CNT agglomeration at high filler loadings [45,53,54]. However, the deviations are below 5% (error < 5%), which are satisfactory for modeling. In other words, the plotted curves by the developed model are the best ones, and the minor deviations can be neglected because they are in the normal range of experimental and calculation errors.

The best level of *σ*_0_ for the current predictions is 1 S/m for all samples. Moreover, the values of (*t*, *z*) are calculated as (2, 0.11), (7, 2.15), (3, 0.51), (22, 0.17), and (3, 0.01) nm for the PDMS/MWCNT, UPE/MWCNT, PVC/MWCNT, PET/MWCNT, and epoxy/SWCNT samples, respectively. The values of *t* are reasonable because they are in a common range for polymer nanocomposites. It should be noted that the value of *z* for CNT nanocomposites differed from the 0.2 to ~13 nm at the aspect ratio (length per diameter) below 1000 [39]. Nevertheless, our developed model based on the tunneling effect can suggest the proper calculations for the conductivity of PCNT.

### 3.2. Tunneling Distance

**Figure 5:**
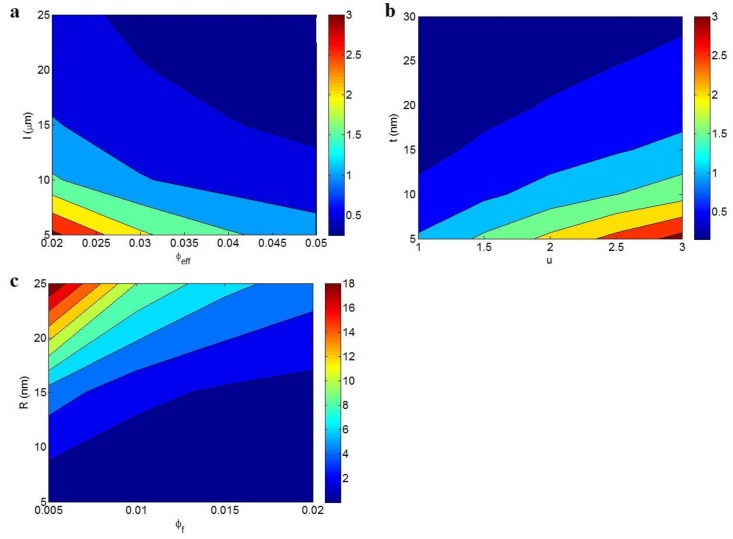
The tunneling distance (Equation (12)) as a function of (**a**) $\varphi_{eff}$ and *l*, (**b**) u and *t*, and (**c**) $\varphi_{f}$ and *R* parameters.

The effects of different parameters on the tunneling distance (*d*) (Equation 12) are observed in Figure 5. Figure 5a shows the impacts of $\varphi_{eff}$ and *l* parameters (effective filler fraction and CNT length, respectively) on *d*. The shortest tunneling distance of about 0.25 nm is obtained by $\varphi_{eff}$ > 0.035 and *l* > 18 μm, while the largest *d* of 3 nm is observed at $\varphi_{eff}$ = 0.02 and *l* = 5 μm. Accordingly, both the effective volume fraction and the CNT length inversely affect the tunneling distance.

A high $\varphi_{eff}$ is representative of high filler concentration, thin nanotubes, and a thick interphase. In this condition, a high number of thin nanotubes enclosed by a thick interphase are incorporated in the nanocomposites, which significantly lessen the distance between neighboring nanoparticles. Thus, the inverse relation between effective filler fraction and tunneling distance is true. In addition, long nanotubes have short inter-particle distances because they occupy a large space in the nanocomposite and produce strong particle–particle interactions. Accordingly, the opposite relation between tunneling distance and CNT length is also logical, which supports our developed equation.

Figure 5b also presents the different roles of the *u* and *t* parameters in the tunneling distance at average $\varphi_{f}$ = 0.01, *R* = 10 nm, and *l* = 10 μm. The tunneling distance is enhanced by high waviness and a thin interphase, whereas a short tunneling distance is produced by less waviness and a thick interphase. Therefore, it is important to reduce the CNT waviness and thicken the interphase to attain a short tunneling distance, which benefits the conductivity of nanocomposites.

The effects of these parameters on the tunneling distance are reasonable because they manage the contacts among nanoparticles. Low waviness indicates straight CNTs in the nanocomposite, which effectively raises the number of contacts and shortens the distance between adjacent nanotubes. On the other hand, a thick interphase shortens the distance between any two nanotubes, because the interphase regions cover the nanotubes. In other words, the conductivity in the interphase zones is greater than in the polymer matrix and less than in the nanoparticles, which can benefit the dimensions of the networks and the conductivity of nanocomposites. Thus, the negative correlation between the tunneling distance and the interphase thickness is attributed to the positive contribution of the interphase regions to the conductivity of nanocomposites as well as nanoparticles.

The tunneling distance at different ranges of $\varphi_{f}$ and *R* and average *l* = 10 μm, *t* = 10 nm, and *u* = 1.3 are plotted in Figure 5c. These parameters considerably affect the tunneling distance because they change it from 0 to 18 nm. High $\varphi_{f}$ and low *R* obtain a short tunneling distance, whereas the tunneling distance grows by decreasing $\varphi_{f}$ and enhancing *R*. As a result, a short tunneling distance is produced by a high filler concentration and small filler radius. As observed, $\varphi_{f}$ = 0.005 and *R* = 25 nm result in *d* = 18 nm, while $\varphi_{f}$ > 0.013 and *R* < 15 nm produce *d* ≈ 0 nm. A high filler concentration creates a large number of nanotubes in the nanocomposite and, obviously, the space between neighboring nanotubes declines significantly, which diminishes the tunneling distance. Additionally, the number of nanotubes and their radius show an adverse relation, because a small *R* produces a high number of nanotubes in a unit volume. Furthermore, thinner nanotubes yield more surface area compared to thicker ones, which enhances the interphase regions [55]. Accordingly, thinner nanotubes can reduce the space between nanotubes, which creates a short tunneling distance between nanotubes. These observations endorse the different influences of the $\varphi_{f}$ and *R* parameters on the tunneling distance based on the developed equation.

### 3.3. Percolation Threshold

**Figure 6:**
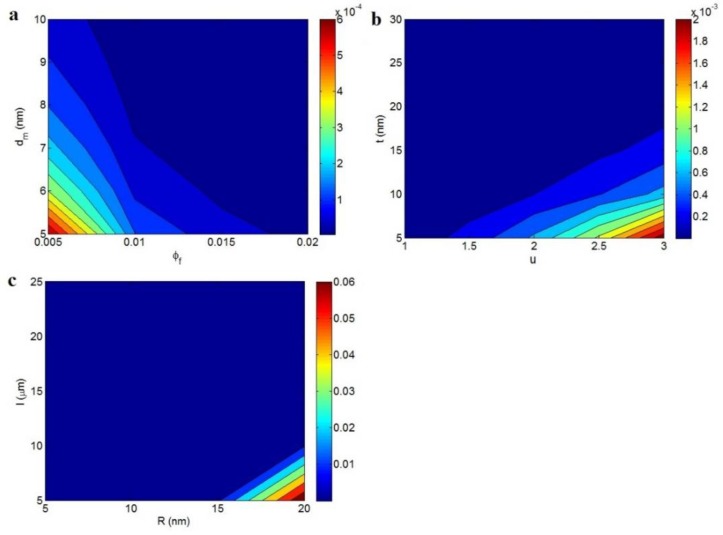
The variation of percolation threshold at different ranges of (**a**) $\varphi_{f}$ and *d_m_*, (**b**) *u* and *t*, and (**c**) *R* and *l* parameters.

Figure 6 exhibits the impacts of various parameters on the percolation threshold based on Equation (13), assuming the tunneling mechanism. The roles of the $\varphi_{f}$ and *d_m_* parameters in $\varphi_{p}$ at average levels of the other parameters are plotted in Figure 6a. It is found that both $\varphi_{f}$ and *d_m_* parameters inversely control the percolation threshold, i.e., the high levels of these parameters reduce the percolation threshold. The $\varphi_{p}$ level of ~0 is obtained at $\varphi_{f}$ > 0.01 and *d_m_* > 7 nm, while $\varphi_{p}$ reaches 0.0006 at $\varphi_{f}$ = 0.005 and *d_m_* = 5 nm. As a result, a desirable percolation threshold is obtained by the highest values of filler fraction and maximum tunneling distance. 

When the filler concentration grows, the tunneling spaces between nanotubes condense and the nanotubes show a high number of contacts. Therefore, a higher filler concentration produces a better condition for percolating and networking, which decreases the percolation threshold. Furthermore, *d_m_* shows the maximum tunneling distance in which the tunneling effect occurs. In other words, the percolation threshold and formation of conductive networks occur when the tunneling distance is smaller than *d_m_* [31]. Thus, a high *d_m_* value permits the distant nanotubes to participate in the conductive networks, producing a low percolation threshold. In summary, our suggested equation properly predicts the converse effects of both $\varphi_{f}$ and *d_m_* parameters on the percolation threshold.

Figure 6b also reveals the effects of *u* and *t* parameters on the percolation threshold at average $\varphi_{f}$ = 0.01, *R* = 10 nm, *l* = 10 μm, and *d_m_* = 7 nm. The *d_m_* value was reported in the literature as ranging from 1.8 to 10 nm [31,42]; therefore, we used an average value of *d_m_* = 7 nm for our calculations. The highest percolation is observed at high *u* and low *t* values, but $\varphi_{p}$ significantly falls as *u* reduces and *t* grows. The most desirable $\varphi_{p}$ is obtained as ~0 at *u* < 1.5 or *t* > 17 nm, which demonstrates the benefits of less CNT waviness and a thick interphase in the percolation level. Less waviness or more straightness depicts the extraordinary effective length of nanotubes. Hence, a low percolation threshold is expected in this condition because the longer nanotubes are more likely to network. In addition, a thick interphase around nanotubes shortens the distance between adjacent nanotubes, raising the probability of percolating and networking. In fact, the interphase areas can establish conductive networks before the definite linking of nanotubes [56]. Accordingly, the positive effects of low CNT waviness and a thick interphase on the percolation threshold confirm our new equation.

The influences of the *R* and *l* parameters on the percolation threshold are also observed in Figure 6c at average $\varphi_{f}$ = 0.01, *t* = 10 nm, *u* = 1.3, and *d_m_* = 7 nm. A high percolation threshold of 0.06 is observed at *R* = 20 nm and *l* = 5 μm, but the percolation threshold declines to ~0 at *R* < 15 nm or *l* > 10 μm. As a result, a small *R* value and a large *l* value significantly lower the percolation threshold, demonstrating the positive effects of thin and long nanotubes on the percolation threshold.

Previous studies have reported the identical influences of these parameters on the geometric percolation threshold [36,38], which confirm that the tunneling effect does not change the effects of the CNT dimensions on the percolation threshold. Thin and long nanotubes yield a high aspect ratio, which moves the percolation threshold to smaller filler concentrations. In fact, slim and long nanotubes increase the effective number and surface areas of nanofillers in a nanocomposite, decreasing the particle–particle distance, due to more inter-contacts between them and a thick interphase. As a result, the percolating and networking of thinner and longer nanotubes are easier than with thicker and shorter ones. In summary, the dependencies of the percolation threshold on the *R* and *l* parameters are reasonable, which demonstrate the correctness of the developed equation.

## 4. Conclusions

We simulated the electrical conductivity, tunneling distance, and percolation threshold of PCNT at various CNT concentrations. Good agreements between experimental results and estimations (deviation less than 5%) confirmed the predictability of the presented model. CNT length and characteristic tunneling length directly influenced the conductivity. High CNT waviness and a thin interphase generally resulted in an insulated nanocomposite, whereas less waviness and a thick interphase produced high conductivity. A high concentration of thin CNTs also caused high conductivity. The nanocomposite conductivity changed from 0 to 0.9 S/m at different values of all the studied parameters using the developed equation. A high filler volume fraction, long and thin CNTs, low CNT waviness, and a thick interphase produced a short tunneling distance, which is desirable for tunneling conductivity. The developed equation yielded minimum and maximum tunneling distances of 0 and 18 nm, respectively. Furthermore, the highest filler concentration, the longest tunneling distance, the least CNT waviness, the thickest interphase, and the highest aspect ratio caused the lowest percolation threshold, which advantageously controls the conductivity. Our calculations indicated the percolation threshold to be in the range of 0 to 0.06. Among the studied parameters, the concentration and dimensions of the CNTs had the most significant effects on the percolation threshold, tunneling distance, and conductivity of nanocomposites.
